# Occupational Injury Claims Related to Patient Lifting and Moving in a Safety-Oriented Emergency Medical Services Agency

**DOI:** 10.7759/cureus.10404

**Published:** 2020-09-12

**Authors:** Kyle A Fratta, Matthew J Levy, James M Brothers, Gamaliel D Baer, Becca Scharf

**Affiliations:** 1 Emergency Medicine, Campbell University, Lillington, USA; 2 Emergency Medicine, Johns Hopkins University School of Medicine, Baltimore, USA; 3 Office of the Medical Director, Howard County Department of Fire and Rescue Services, Marriottsville, USA; 4 Emergency Medical Services, Howard County Department of Fire and Rescue Services, Ellicott City, USA; 5 Emergency Medical Services, Howard County Department of Fire and Rescue Services, Marriottsville, USA

**Keywords:** ems provider lifting injuries, ems provider occupational injuries, emergency medical services, occupational injury

## Abstract

Background: Patient lifting injuries remain a significant hazard to Emergency Medical Services (EMS) providers despite preventative and mitigative strategies.

Objective: To better characterize the nature of occupational injury involving patient and stretcher handling.

Methods: A retrospective review of existing de-identified claims data was performed for the study period of January 1, 1999, through December 31, 2017. Independent reviewers analyzed each claim to determine if the claim was related to lifting or moving a patient. Any discrepancies between the two reviewers were analyzed by a third reviewer.

Results: Eighty-two claims were identified as resulting from lifting or maneuvering patients. Fifty-two of these injuries (63.4%) resulted in at least one lost workday (LWD). Strains and sprains accounted for the majority of injuries with 63.4% (n=52) and 18.3% (n=15) respectively. Forty-two (51.2%) of these reports occurred when the provider was moving a patient, not involving a stretcher, while 37.8% (n=31) occurred due to lifting or maneuvering a stretcher with or without a patient.

Conclusion: While the overall incidence of lifting injuries was less than reported in other occupational health data series, these injuries continue to occur, and cause significant operational and fiscal impact for EMS systems. This occurrence is despite advances in engineering controls and the organizational embracement of a culture of safety that focuses on risk identification and mitigation. Understanding the types of lifting/moving injuries, circumstances surrounding the injury, and contributing factors will help to maintain a heightened awareness of potential injuries associated with EMS work, and opportunities to reduce them.

## Introduction

Prehospital Emergency Medical Services (EMS) are an inherently dangerous profession with a multitude of occupational hazards. These hazards include exposures, falls, motor vehicle collisions, violence, and overexertion [1]. These physical injuries occur more frequently in EMS than in other industries, including non-EMS fire rescue agencies, with a reported injury rate of 34.6 per 100 full-time employees (FTE) [2]. These injuries result in a lost workday (LWD) rate approximately three-times higher than that of private-industry [3].

Over-exertion injuries are the most frequent type of bodily injury in EMS [1]. These include strains and sprains to muscles and joints as a result of a single lift or exertion that exceeds that muscle group's strength. EMS workers are at a higher risk of over-exertion injuries [2]. Risk factors such as heavy loads, lifting in awkward positions, and working in extreme temperatures are a regular part of providing emergency care in the field [4]. Forty percent of all overexertion injuries in EMS were as a direct result of lifting, and over half of sprain and strain injuries were related to interactions with patients [1]. EMS providers are frequently required to lift patients of varying weights onto stretchers, and subsequently, lift and then maneuver these stretchers to and from the ambulance, and again lift the person onto an emergency department stretcher once at the hospital [4].

In an effort to decrease the high rate of injury and personal risk, Fire/Rescue and EMS departments have implemented a variety of cultural, educational, and ergonomic prevention and mitigation strategies. Some of the solutions implemented in the EMS system studied herein aim at ensuring adequate baseline physical readiness of employees via pre-employment physical agility tests and medical examinations. While others aim at maintaining physical readiness longitudinally, these include providing physical fitness equipment at EMS stations for use while on duty as well as required yearly medical examinations. In addition to providing fitness equipment and medical evaluations, ergonomic interventions such as powered patient stretchers and powered stretcher loading devices have become more widespread to specifically address lifting injuries associated with stretcher and patient handling.

Injuries related to patient lifting and moving can occur while transferring patients both to and from EMS stretchers and while actually operating the EMS stretchers. Efforts to help prevent injuries associated with lifting and operating EMS stretchers include the implementation of powered lifting stretchers and loading systems. These devices reduce the physical workload on EMS workers by utilizing powered technologies to raise and lower the patient stretcher and to load and unload the stretcher from the ambulance. Several studies have demonstrated the biomechanical stresses of loading a variety of stretchers and their effect on EMS work-place injuries. Sommerich et al. found that powered stretchers drastically reduced muscle maximum voluntary exertions in the trunk and upper body during lifting and lowering activities [5]. Prairie et al. found that powered stretchers alone were not enough to make the loading and unloading tasks safe, since one provider still had to bear the weight of the stretcher as it was taken in and out of the ambulance [6]. Despite this, the implementation of powered stretchers alone in a large EMS system has been shown to successfully decrease the rate of injury, especially among injuries relating to stretcher use [4].

The primary objective of this study is to describe the prevalence and details of lifting-related injuries reported in a moderate-sized safety-oriented Fire/Rescue EMS Agency. A secondary aim of this study is to identify “high-cost” injuries and to expound on the mechanisms and details of these injuries.

## Materials and methods

This study consisted of secondary analysis and a retrospective review of existing de-identified claims data for the study period of January 1, 1999 through December 31, 2017. This information was organized in a Microsoft Excel (Redmond, Washington, USA) database. Two independent reviewers analyzed each claim to determine if the claim was related to lifting or moving a patient or equipment. Any discrepancies between the two reviewers were analyzed by a third reviewer. The claims data were from a single moderate-sized sub-urban Fire/Rescue EMS agency. This agency employs approximately 500 career uniformed employees and 200 volunteer providers. Of the uniformed employees, 87% are male and 13% are female. The Howard County Department of Fire and Rescue Services (HCDFRS) responds to an average of 28,000 EMS calls per year. The agency serves a county of approximately 300,000 people over 251 square miles. This study was deemed to be exempt from non-human subjects research under provisions of the Code of Federal Regulations Revised Common Rule 46.104(d) (1-8).

## Results

There were 1,186 injuries reported during the 18-year study period. Eighty-two (6.9%) of these injuries were identified as resulting from lifting, moving, or maneuvering a patient or as a direct result of patient care, not including violence. The average age among these 82 EMS providers was 44.7 years (SD=8.9), ranging from 24-68 years. Seventy-three percent of those reporting an injury were male (n=60), 20.7% were female (n=17), and 6.1% did not have their sex reported (n=5). Fifty-two of these injuries (62.6%) resulted in at least one LWD with a range of 0-198 days lost. Nineteen injuries did not result in any LWD and eleven injuries did not have a recorded return to work date. In total, 707 days were lost with an average of 10.0 LWDs (SD=28.4) per incident.

Strains and sprains accounted for the majority of injuries with 63.4% (n=52) and 18.3% (n=15) respectively (Table 1). The three most commonly injured body areas were the back (31.7%, n=26), followed by the wrist (22%, n=18), and the abdomen/groin (9.8%, n=8) (Table 2). The most prevalent injury type was “strain or injury by lifting” with a reported 54.8% (n=47), although there was an overlap of injury types in the data reviewed (Table 3). 

**Table 1:** Type of Injury Incidence

Type of Injury	Incidence
Strain	52
Sprain	15
Not Specified	5
Hernia	4
Laceration	2
Contusion	2
Amputation	1
Crush	1

**Table 2:** Area Injured Incidence

Area Injured	Incidence
Back	26
Wrist (s)	18
Abdomen Including Groin	8
Knee	4
Shoulder(s)/Arm(s)	5
Hand and Fingers	4
Leg(s)/Feet	4
Neck	2
Multiple Body Parts	1
Not Specified	10

**Table 3:** Accident Type Incidence

Accident Type	Incidence
Strain or injury by lifting	47
Lifting	9
Not specified	6
Overexertion	5
Strain or injury by pushing or pulling	3
Caught in, under, or between – misc.	2
Twisting	2
Bodily reaction	1
Fall/slip on ice/snow	1
Falling or flying object	1
Holding or carrying	1
Injured by detainee, public, etc.	1
Object lifted or handled	1
Slip (not fall) involuntary bodily reaction	1
Struck by flying, falling or moving object	1
Struck by, against, etc.	1
Training/exercising	1

Forty-two (51.2%) of these injuries occurred when the provider was lifting, transferring, or carrying a patient, not involving a stretcher. Thirty-one (37.8%) of these injuries occurred while lifting, loading, or unloading a stretcher with or without a patient on it, seven (8.5%) occurred while caring for a patient, and two (2.4%) occurred while lifting equipment, not including the stretcher. One incident occurred when a provider was operating a powered stretcher resulting in a partial finger amputation.

A total incurred cost of $486,113.96 was attributed to these 82 reported injuries, not including LWDs and related salary/overtime costs. Total injury costs ranged from $0.00 to $82,354.56 with an average cost of $5,928.22 (SD=$12,764.45) and 80 of the 82 injuries incurring some cost. Total yearly costs ranged from $441.69 to $85,819.71 (Table 4). Eighteen of the reports were identified as costing over $5,000.00. Providers who were injured in these high-cost injuries were on average 45.3 years old (SD=9.7) and majority male (66.7%). Of these high-cost injuries the most common cause was lifting or transferring a patient, not involving a stretcher at 55.6% (n=10), followed by lifting involving a stretcher 22.2% (n=4), maneuvering a stretcher 11.1% (n=2), and an injury caused by the stretcher’s operation 5.6% (n=1). 

**Table 4:** Annual Costs * Note: No standard deviation reported for FY 2010 as only one charge reported in that year

FY	Average Cost (SD)	Total Cost
2000	$555.14 ($669.50)	$3,330.82
2001	$952.20 ($1,647.54)	$6,665.41
2002	$5,976.17 ($12,027.75)	$29,880.87
2003	$11,075.43 ($12,360.49)	$44,301.71
2004	$5,719.98 ($10,947.81)	$22,879.93
2005	$775.69 ($494.53)	$2,327.07
2006	$21,454.93 ($40,612.36)	$85,819.71
2007	$9,706.03 ($16,105.32)	$67,942.21
2008	$2,183.48 ($1,795.19)	$8,733.90
2009	$7,164.07 ($10,344.79)	$21,492.21
2010	$441.69 (-)	$441.69
2011	$2,832.81 ($5,515.41)	$19,829.68
2012	$4,495.66 ($9,322.60)	$26,973.94
2013	$5,307.07 ($6,919.65)	$10,614.14
2014	$2,233.86 ($4,156.84)	$13,403.14
2015	$627.45 ($584.97)	$1,254.89
2016	$5,831.75 ($5,794.62)	$29,158.74
2017	$25,767.71 ($33,194.54)	$51,535.41
2018	$9,882.12 ($10,173.35)	$39,528.49
Total	$5,928.22	$486,113.96

Among the high-cost injuries, strains were most common with 61.1% (n=11), followed by hernias 16.7% (n=3), sprains 11.1% (n=2), contusions 5.6% (n=1), and the amputation of a finger-tip 5.6% (n=1). The body areas most commonly affected in the high-cost injuries subset included the abdomen/groin with 27.8% (n=5) and back 27.8% (n=5).

## Discussion

EMS providers have one of the highest rates of injury and illness of all occupations [7]. Often these injuries occur due to overexertion while moving or caring for a patient affecting a wide variety of bodily areas. In response to these bodily injuries, numerous ergonomic and systems interventions have been developed. This study analyzed 18 years of retrospective injury claim data in an attempt to describe injuries in a medium-size sub-urban Fire/Rescue EMS Service.

In the 18 years that were analyzed in this study, 1,186 total claims were made and of those only 82 (6.9%) were as a result of lifting or maneuvering patients or equipment. This equates to an average of just 4.6 lifting related injuries per year. In an analysis of a combined Fire/Rescue and EMS service of comparable size to the studied service, 720 workers' compensation events were documented in just a 29 month-span [8]. Five-hundred and twenty (72.2%) of these injuries were defined as minor traumatic events. These injuries were mostly associated with axial musculoskeletal strains and extremity injuries. As minor traumatic events such as sprains and strains make up a majority of injuries in public service, addressing these might have the largest effect on the quality of life and productivity of EMS workers [8].

The HCDFRS has implemented several ergonomic and systems interventions in an attempt to address lifting related provider injuries. Pre-employment base-line physical agility testing was done using the Candidate Physical Abilities Test© (CPAT) in 1999. Yearly medical examinations were made mandatory for all career and operational volunteer personnel in 1987. Additionally, the culture of safety includes yearly safety training both online and in-person, strict reporting of injuries, and a proactive mindset in addressing injuries and near-misses. Yearly training in proper lifting technique and safety is incorporated into emergency medical technician (EMT) and paramedic refresher courses. Additionally, the department participates in National Safety Stand-Down, a workplace safety initiative from the Occupational Safety and Health Administration (OSHA), to specifically address fall prevention and fall hazards [9]. Near-miss safety incidents are recorded via tracking software from Industry Safe© (Philadelphia, PA, USA) and reporting is mandatory for Safety Officers. If an injury does occur, reporting is mandatory for all department members following OSHA guidelines. Besides proactive health-conscious interventions, HCDFRS also implemented engineering-based solutions such as hydraulically powered patient stretchers and stretcher loading devices in September of 2013 to decrease the mechanical strain on providers.

## Conclusions

Prehospital EMS represents a dangerous profession with high potential for occupational injury. Previous studies have shown that over-exertion/musculoskeletal injuries are the most frequent type of injury in EMS. In this longitudinal, retrospective analysis of 18 years of a medium-sized single EMS system’s occupational injury claims data, we found that while the overall incidence of such injuries was less than reported in similar data series, these injuries continue to occur, and are with significant operational and fiscal impact. Although lower than reported in other series, this occurrence is despite advances in engineering controls and the organizational embracement of a culture of safety that focuses on risk identification and mitigation. Understanding the types of lifting/moving injuries, circumstances surrounding the injury, and contributing factors will help continue to maintain a heightened awareness of the dangers associated with EMS work, and opportunities to reduce them. Further prospective research is needed to better determine the efficacy of individual interventions in addressing this issue. Additional studies tracking and defining injuries in EMS will also be necessary to keep up with the ever-evolving nature of this profession. With this information, Fire/Rescue and EMS agencies may be better equipped to understand some aspects of occupational injury involving patient and stretcher handling.
